# Prolactinomas in women of reproductive age: a proposed reproductive-intent-based framework for diagnosis and management

**DOI:** 10.3389/fendo.2026.1946204

**Published:** 2026-09-22

**Authors:** Rui Sun, Xuxu Xu, Shepeng Wei

**Affiliations:** 1 Fuyong People’s Hospital, Shenzhen, China; 2 Department of Neurosurgery, Yangpu District Shidong Hospital of Shanghai, Shanghai, China; 3 Shidong Hospital, University of Shanghai for Science and Technology, Shanghai, China

**Keywords:** dopamine agonists, fertility, hyperprolactinemia, pregnancy, prolactinoma

## Abstract

Prolactinomas are the most common functioning pituitary tumors in women of reproductive age and can affect fertility, gonadal and skeletal health, and vision. Management becomes particularly complex when reproductive plans intersect with diagnostic uncertainty, tumor anatomy, pregnancy-related enlargement, and long-term treatment decisions. This focused narrative Mini Review synthesizes current guidelines, consensus statements, systematic reviews, and clinically informative cohort studies and proposes a reproductive-intent-based framework for organizing individualized care. Adequate diagnostic confidence is required before pathway assignment; patients may then enter one of three proposed dynamic management states: fertility-driven, endocrine-stabilization, or surveillance-dominant. Tumor-related risk—including optic-apparatus proximity, documented growth, apoplexy, and clinically important dopamine agonist resistance or intolerance—acts as a cross-cutting safety modifier. Dopamine agonists remain first-line therapy for most patients, with surgery considered for selected anatomically favorable tumors or when medical treatment is ineffective or poorly tolerated. Preconception care aims to restore ovulation and establish anatomical safety; pregnancy surveillance is predominantly symptom-driven; and postpartum reassessment should precede treatment resumption. Persistent hyperprolactinemia alone does not necessarily justify therapy without clinically meaningful hypogonadism, symptoms, or tumor progression. In women not seeking pregnancy, treatment intensity should reflect gonadal status, symptoms, skeletal consequences, and tumor risk. This framework does not replace guideline-based recommendations or constitute a validated clinical classification; rather, it makes management priorities and transitions explicit and requires prospective validation.

## 1 Introduction

Prolactinomas are the most common functioning pituitary tumors in women of reproductive age and a major endocrine cause of anovulatory infertility and hypogonadism through suppression of hypothalamic gonadotropin-releasing hormone pulsatility and downstream gonadotropin secretion (1–3). Throughout this review, “prolactinoma” denotes the established clinical diagnosis, whereas “lactotroph pituitary neuroendocrine tumor (PitNET)” is reserved for contemporary WHO histopathological classification (1, 4). Dopamine agonists, principally cabergoline and bromocriptine, are first-line therapy for most patients; cabergoline is generally preferred for greater efficacy and better tolerability (1, 3). Dose escalation may improve partial responses (5), whereas long-term control and successful withdrawal depend on tumor burden and treatment characteristics (1, 6).

Management becomes more complex when reproductive goals intersect with tumor risk. Pregnancy causes physiological pituitary enlargement (7). Prolactinomas may rarely be complicated by apoplexy, including microprolactinomas (8), while macroprolactinomas near the optic apparatus carry greater risks of clinically significant enlargement and visual compromise (1, 9, 10). Evidence on inadvertent early-gestational dopamine agonist exposure is reassuring overall (10–12). For women not seeking pregnancy, priorities include gonadal function, symptom and tumor control, and prevention of hypoestrogenic bone loss (1, 2, 13). Existing guidance already individualizes care by anatomy, symptoms, treatment response, and reproductive stage (1, 2, 9). This Mini Review therefore does not propose a new clinical classification or replace guideline-based recommendations. Rather, after diagnostic confidence is established, it organizes existing guidance into three dynamic, reproductive-intent-based pathways—fertility-driven, endocrine-stabilization, and surveillance-dominant—making treatment priorities and transitions explicit, with tumor risk as a cross-cutting safety modifier. These proposed, unvalidated management states allow movement as reproductive goals, gonadal function, symptoms, and anatomy evolve.

## 2 Review approach

PubMed/MEDLINE was last searched on August 25, 2026, using combinations of “prolactinoma,” “hyperprolactinemia,” “pregnancy,” “fertility,” “dopamine agonist,” “surgery,” and “postpartum.” Guidelines and consensus statements were prioritized, followed by systematic reviews and clinically informative cohort studies; reference lists were also screened. This was a focused narrative search, not a systematic review.

## 3 Definition, anatomical classification, and diagnostic certainty

“Prolactinoma” denotes a clinical diagnosis of a pituitary lesion causing pathological PRL secretion (1, 2); “lactotroph pituitary neuroendocrine tumor (PitNET)” is used only for WHO histopathological classification (4). Because tissue confirmation is uncommon, diagnosis integrates reproducible hyperprolactinemia, exclusion of secondary causes and laboratory confounders, and a compatible lesion on initial sellar MRI (1, 2). Mild or discordant elevations require repeat low-stress sampling; neither PRL nor MRI is independently diagnostic. PRL is reported in ng/mL (1 ng/mL = 1 μg/L); mIU/L values require assay-specific conversion factors.

Guideline-supported anatomical categories are microprolactinoma (<10 mm), macroprolactinoma (≥10 mm), and giant prolactinoma (generally ≥40 mm with substantial extrasellar extension) (1, 2). Size informs pregnancy risk, treatment intensity, surveillance, and withdrawal success, but must be interpreted with suprasellar extension, cavernous sinus invasion, growth pattern, cystic components, and proximity to the optic apparatus (1, 2). In the proposed framework, these features are cross-cutting safety modifiers, not reproductive-intent categories.

PRL magnitude modifies diagnostic probability but is not an absolute cutoff. The 2023 Pituitary Society consensus considers >200 ng/mL more suggestive of prolactinoma in an appropriate clinical-radiological context (1); earlier guidance associates >250 ng/mL with prolactinoma and >500 ng/mL strongly with macroprolactinoma (2, 3). These are interpretive aids, not universal diagnostic or treatment thresholds. In a retrospective series of 114 established prolactinomas, approximately 204 ng/mL distinguished micro- from macroprolactinomas with high sensitivity and specificity (14), but did not distinguish prolactinoma from non-functioning adenoma or other causes. Values <200 ng/mL show greatest overlap with microprolactinoma, stalk effect, macroprolactinemia, medications, physiological factors, and systemic disease (1–3).

An unexpectedly modest PRL concentration in a large sellar mass requires repeat testing after serum dilution to exclude the high-dose hook effect (1, 2). Macroprolactin testing is appropriate when biochemical findings are asymptomatic or clinically discordant. During dopamine agonist therapy, PRL reduction alone is nondiagnostic because stalk-effect hyperprolactinemia may also improve; measurable tumor shrinkage more strongly supports lactotroph origin but must be interpreted with baseline PRL, morphology, symptoms, and longitudinal imaging (1, 2).

Within this framework, diagnostic confidence is a pre-pathway eligibility requirement, not a fourth pathway. Patients with unexplained hyperprolactinemia, a compatible lesion, and biochemical–anatomical concordance may enter reproductive-intent-based pathway assignment. Modest PRL elevation, equivocal imaging, or marked discordance instead warrants repeat biochemistry, assessment for assay interference, expert MRI review, and/or longitudinal reassessment. This author-proposed diagnostic gate is an operational component, not an established clinical classification.

## 4 A proposed reproductive-intent–based management framework

Existing guidance already individualizes prolactinoma care by anatomy, symptoms, gonadal function, treatment response, reproductive plans, pregnancy, and postpartum status (1, 2, 9). This framework is therefore organizational, not a new disease classification: diagnostic confidence precedes assignment; current reproductive intent and the dominant clinical objective determine the pathway; tumor risk is a cross-cutting safety modifier; and transitions are explicit. The labels, allocation criteria, and transition rules are author-proposed and require prospective validation.

Adequate diagnostic confidence is required for assignment. Patients with modest or fluctuating PRL elevation, equivocal MRI, or biochemical–radiological discordance remain in pre-pathway diagnostic reassessment until secondary causes, assay interference, and alternative sellar pathology are sufficiently evaluated. Diagnostic uncertainty is distinct from the surveillance-dominant pathway, which applies only to established low-risk prolactinoma.

After this gate, the fertility-driven pathway applies to near-term pregnancy and prioritizes ovulation and preconception anatomical safety. The endocrine-stabilization pathway applies when pregnancy is not intended but treatment is required for clinically meaningful hypogonadism or menstrual dysfunction, bothersome symptoms, hypoestrogenic or skeletal consequences, or tumor control. The surveillance-dominant pathway is reserved for established low-risk disease with preserved gonadal function, no important symptoms or hypoestrogenic consequences, stable anatomy, and no optic threat. These are dynamic management states, not fixed phenotypes.

Tumor risk modifies every pathway. New visual symptoms or field deficits, optic-apparatus proximity or compression, documented growth, apoplexy, or clinically important dopamine agonist resistance or intolerance should prompt reassessment and, when appropriate, medical or surgical escalation regardless of pathway (1, 2, 9). An isolated PRL increase instead warrants confirmation and integrated clinical and radiological reassessment, not automatic escalation. **Table 1** summarizes operational criteria and evidence; **Figure 1** depicts the pathways and transition triggers.

**Table 1:** Proposed reproductive-intent–based management pathways: entry criteria, guideline-consistent management, and author-proposed transitions.

Pathway	Entry criteria and objective	Guideline-consistent management	Author-proposed transitions or escalation
Fertility-driven pathway (A)	Active near-term pregnancy intent; preconception anatomy assessed. Objective: restore ovulation and ensure anatomical safety before conception.	Use dopamine agonist therapy to normalize PRL, restore ovulation, and reduce tumor burden; cabergoline is generally preferred before conception (1, 2). Macroadenomas, invasive disease, or optic-apparatus proximity require adequate shrinkage or stability before conception (9, 10). Consider surgery for dopamine agonist intolerance or resistance or when timely anatomical control is needed (1). Evaluate other infertility factors if conception does not follow restored ovulation (10).	Pregnancy activates pregnancy-specific management. If pregnancy plans are deferred or completed, transition to pathway B if an endocrine or tumor-control indication persists, or to pathway C if gonadal function is preserved and anatomy remains low-risk and stable. Inadequate biochemical or tumor response, visual compromise, or growth activates tumor-safety escalation.
Endocrine-stabilization pathway (B)	No current pregnancy intent plus clinically meaningful hypogonadism or menstrual dysfunction, bothersome symptoms, hypoestrogenic or skeletal consequences, or need for tumor control. Objective: preserve gonadal function, control symptoms, and maintain tumor stability.	Use dopamine agonist therapy when indicated and assess gonadal and bone health (1, 13, 15). After biochemical and anatomical control, maintain the lowest effective dose (1). Consider dose reduction or withdrawal only when guideline criteria are met (1) and structured follow-up is feasible (16–18).	New pregnancy intent prompts transition to pathway A. Sustained biochemical control, preserved gonadal function, no important symptoms, and stable low-risk anatomy may permit transition to pathway C, including after supervised dose reduction or withdrawal. Recurrent hypogonadism, relevant symptoms, or tumor progression requires renewed or intensified treatment; dopamine agonist resistance or intolerance may prompt surgical reassessment.
Surveillance-dominant pathway (C)	No current pregnancy intent; preserved gonadal function; no bothersome symptoms or meaningful hypoestrogenic consequences; and stable low-risk anatomy without optic threat. Objective: avoid unnecessary treatment while confirming stability.	Perform serial clinical and endocrine review with PRL measurement. Individualize follow-up MRI according to initial tumor size, prior behavior, treatment history, and new clinical or biochemical concerns. A confirmed PRL rise prompts reassessment but does not mandate treatment without meaningful endocrine consequences or tumor progression (1, 2).	New pregnancy intent prompts transition to pathway A. Hypogonadism, menstrual dysfunction, bothersome symptoms, or skeletal consequences prompt transition to pathway B. Tumor growth, visual symptoms, or optic-apparatus threat activates tumor-safety escalation. New biochemical–radiological discordance returns the patient to diagnostic reassessment.

The pathway labels, entry criteria, and transition rules are author-proposed; management strategies in the third column derive from guidelines, consensus statements, and published evidence. Adequate diagnostic confidence is required before entry into any pathway, and the tumor-safety escalation rule applies across all pathways. MRI, magnetic resonance imaging; PRL, prolactin.

**Figure 1:**
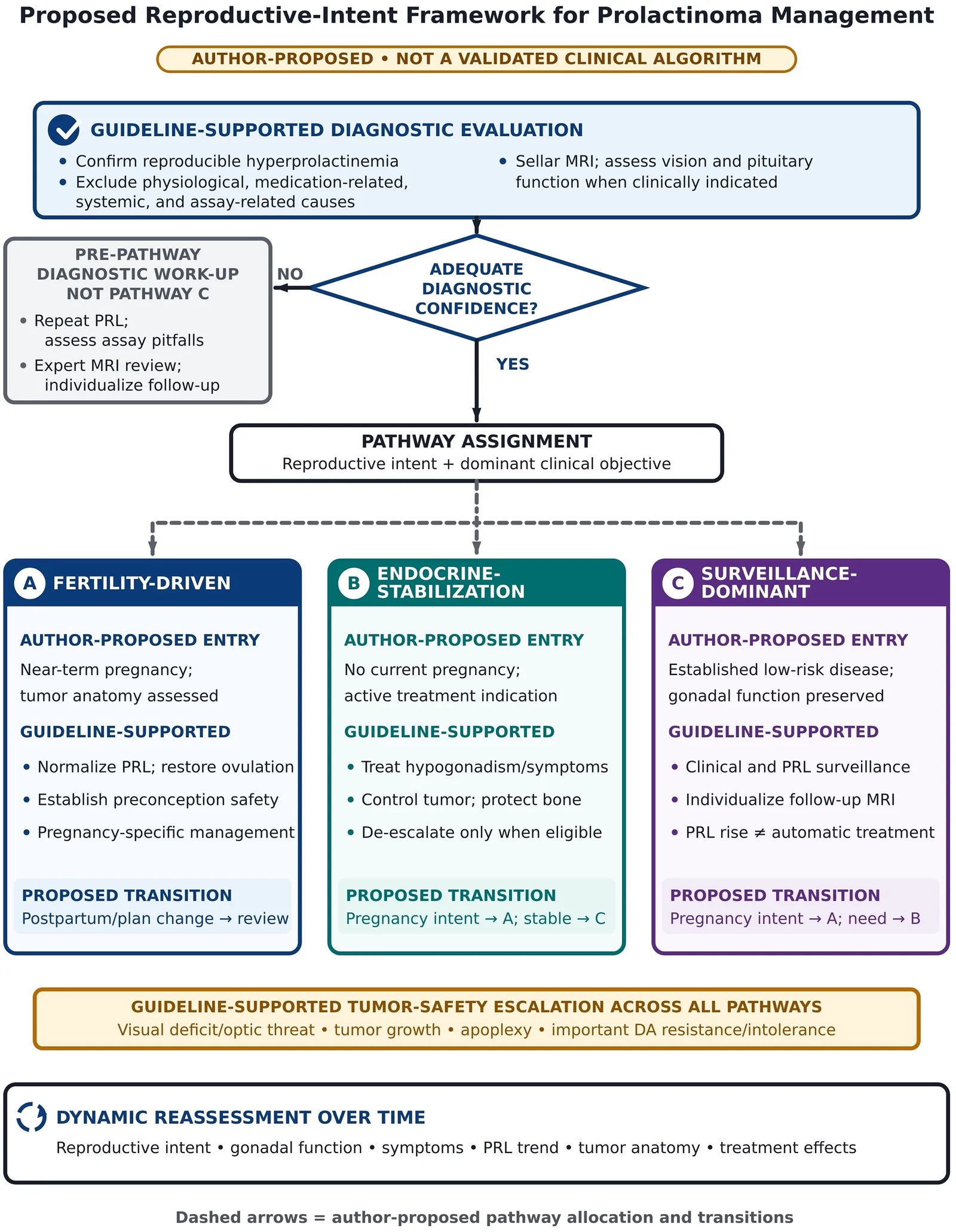
Proposed conceptual reproductive-intent–based management framework for women with prolactinoma. Adequate diagnostic confidence precedes assignment; patients with uncertainty remain in pre-pathway reassessment. Patients then enter fertility-driven **(A)**, endocrine-stabilization **(B)**, or surveillance-dominant **(C)** management states according to reproductive intent and the dominant clinical objective. Tumor risk modifies all pathways, and changes in reproductive plans, gonadal function, symptoms, PRL trajectory, or anatomy may trigger transition or escalation. Within the solid pathway boxes, labels distinguish guideline-supported management from author-proposed entry and transition criteria; dashed arrows indicate author-proposed allocation and transitions. This is not a validated clinical algorithm. DA, dopamine agonist; MRI, magnetic resonance imaging; PRL, prolactin.

Pathway assignment should be reconsidered when pregnancy or lactation status, reproductive intentions, gonadal function, symptoms, treatment status, or biochemical and anatomical findings change. Pregnancy activates the pregnancy-specific strategy described below; postpartum is a reassessment point, not an indication for automatic dopamine agonist resumption. Reassignment should reflect current reproductive intent, clinically meaningful recurrence of hypogonadism or symptoms, and tumor persistence or progression—not hyperprolactinemia alone.

This model organizes individualized care and changing priorities but does not override guidelines or multidisciplinary judgment. Prospective studies must determine whether it improves clinical consistency, patient-centered decisions, or reproductive and tumor outcomes.

## 5 Diagnostic evaluation

Biochemical evaluation: Serum prolactin should be measured under low-stress conditions. One clearly abnormal result is usually sufficient; mild or clinically discordant elevations should be repeated, and dynamic stimulation testing is not recommended (1, 2). Before attributing hyperprolactinemia to a prolactinoma, pregnancy or lactation, primary hypothyroidism, renal or hepatic dysfunction, and prolactin-raising medications should be excluded (1, 2). Macroprolactin testing is appropriate when PRL elevation is discordant with symptoms or gonadal status. In a large sellar lesion with unexpectedly modest PRL, the assay should be repeated after serial dilution to exclude the hook effect before attributing the elevation to stalk effect (1–3). Stalk effect generally causes mild-to-moderate hyperprolactinemia, but overlap precludes a single diagnostic cutoff (1, 2).

Imaging: For initial diagnosis, dedicated thin-section contrast-enhanced sellar MRI should define lesion size, suprasellar extension, relationship to the optic apparatus, cavernous sinus invasion, and cystic or hemorrhagic components (1, 19). Small hypoenhancing foci should not be interpreted independently of biochemical findings. During follow-up, MRI should generally be repeated 3–6 months after dopamine agonist initiation for macroprolactinomas; for microprolactinomas, imaging depends on the clinical and biochemical course and may be performed at approximately 1 year or before treatment withdrawal (1). Selected follow-up MRI of a stable, previously documented microadenoma may be performed without contrast (20). During pregnancy, routine MRI is not recommended. New visual-field impairment, cranial-nerve palsy, severe or progressive headache, or concern for tumor growth or apoplexy warrants neuro-ophthalmic assessment and MRI without gadolinium (9).

Visual fields and pituitary function: Baseline automated perimetry is indicated for lesions approaching or impinging the optic chiasm and for any patient with visual symptoms; it should be repeated if visual symptoms develop or tumor enlargement occurs (1, 2). Macroadenomas and other large sellar masses require assessment of morning cortisol, with dynamic testing when indicated, thyroid function, IGF-1, and the gonadal axis. Adrenal insufficiency and hypothyroidism must be corrected before conception, and gonadal-axis status should inform fertility planning (1, 2, 9).

## 6 Preconception care and treatment before pregnancy

Consistent with established guidelines, preconception care aims to normalize prolactin, restore ovulation, establish tumor safety, and establish an individualized monitoring plan (1, 9, 10). Assessment includes menstrual and ovulatory status, prolactin control, dopamine agonist efficacy and tolerability, and recent sellar MRI. Formal perimetry is required when the tumor approaches the optic apparatus, and pituitary function should be assessed in macroadenomas to correct adrenal insufficiency and hypothyroidism before conception (1, 2, 9). These elements are guideline-supported; their sequencing within the fertility-driven pathway is author-proposed.

Dopamine agonist therapy: Cabergoline is generally preferred at the lowest effective dose until pregnancy is confirmed; bromocriptine remains an acceptable alternative (1, 2, 9). Systematic reviews show no clear increase in miscarriage, congenital malformations, or preterm birth after early exposure to either agent, although evidence remains observational (10–12). Fertility may return before cycles fully regularize. If conception does not follow restored ovulation, female and male infertility factors should be evaluated. Counseling should address common gastrointestinal and orthostatic adverse effects and uncommon impulse-control or behavioral changes (1).

Treatment should be titrated to prolactin normalization, ovulation, symptoms, and tumor response rather than a predetermined maximum dose. In macroprolactinomas with suprasellar extension or proximity to the optic chiasm, conception should generally be deferred until adequate biochemical control and tumor shrinkage or stability are achieved, with baseline visual function documented (1, 9, 21). Apparent dopamine agonist resistance requires review of adherence, dose tolerance, assay reliability, and radiological diagnosis, with distinction between incomplete biochemical control and inadequate tumor shrinkage. Cabergoline escalation may overcome partial resistance (1, 5, 6).

Multidisciplinary surgical assessment is appropriate for confirmed dopamine agonist resistance or intolerance, persistent optic risk, inadequate preconception shrinkage, cerebrospinal-fluid leakage, apoplexy with visual compromise, or suspected aggressive growth (1, 2). At an experienced pituitary center, surgery may also be discussed as an alternative first-line option for a well-circumscribed, noninvasive microprolactinoma or selected intrasellar macroprolactinoma when the patient prefers a potentially definitive treatment. Expected remission should be balanced against operative risk and possible new pituitary deficits. Radiotherapy and systemic therapy are reserved for rare aggressive tumors uncontrolled by optimized medication and surgery (1).

Once pregnancy is confirmed, dopamine agonist therapy is ordinarily stopped; continuation may be considered for selected invasive macroprolactinomas, tumors near the optic apparatus, or previous growth after withdrawal (1, 2, 9). According to the 2019 joint position statement, baseline transthoracic echocardiography is recommended before dopamine agonist therapy. For cabergoline doses ≤2 mg/week, repeat imaging is advised at 5 years and every 5 years thereafter if unchanged; annual echocardiography is recommended for doses >2 mg/week (22).

## 7 Management when pregnancy is not currently intended

When pregnancy is not currently intended, pathway B applies when active treatment is justified by clinically meaningful hypogonadism or menstrual dysfunction, bothersome symptoms, hypoestrogenic or skeletal consequences, or tumor-control needs. Pathway C is reserved for established low-risk prolactinoma with preserved gonadal function, minimal symptoms, and stable anatomy. The underlying management strategies are guideline-supported; pathway allocation and transition rules are author-proposed (1, 2, 15).

Selected women with microprolactinomas, preserved gonadal function, minimal symptoms, and no tumor-related threat may be observed (1, 2), with prolactin generally measured every 6–12 months. Repeat MRI should be individualized and obtained after a confirmed prolactin rise, new headache or visual symptoms, pituitary dysfunction, or when imaging would alter management, rather than at fixed intervals (1). Premenopausal women with microprolactinoma-associated hypogonadism who do not desire pregnancy may receive estrogen–progestin therapy instead of a dopamine agonist when tumor shrinkage is unnecessary, with continued clinical and prolactin surveillance (1, 2). This option is inappropriate when macroadenoma-related risk, progression, or mass effect requires tumor-directed treatment.

Dopamine agonist therapy is indicated for clinically meaningful hypogonadism or symptoms and for tumor control; prolactin elevation alone should not determine treatment intensity (1, 2). Bone care should address gonadal recovery, adequate calcium and vitamin D intake, weight-bearing exercise, and additional fracture risks. Bone-density assessment should be individualized according to the duration of hypoestrogenism and overall fracture risk (15).

Withdrawal may be attempted after at least 2 years of sustained normoprolactinemia with substantial tumor reduction (1, 17). Sustained remission is more likely with a low maintenance dose and no visible residual tumor (16, 18). Prolactin should be measured approximately every 3 months during the first year after withdrawal and annually thereafter if stable. Isolated biochemical recurrence should be confirmed and reassessed rather than trigger automatic lifelong treatment. Therapy should be restarted when recurrence is accompanied by clinically meaningful hypogonadism or menstrual dysfunction, bothersome symptoms, or MRI evidence of tumor growth or mass-effect risk; selected asymptomatic, eugonadal women with stable low-risk anatomy may remain under surveillance (16–18). New pregnancy intent requires reassignment to pathway A.

## 8 Management during pregnancy

Surveillance during pregnancy is based primarily on baseline tumor anatomy and preconception control (1, 9, 10). Women should promptly report new or progressive headache, visual blurring, diplopia, or field disturbance. Small intrasellar microprolactinomas with normal pituitary function usually require clinical observation alone, whereas large macroprolactinomas, tumors near the optic chiasm, or lesions incompletely controlled before conception warrant individualized neuro-ophthalmic surveillance (1, 9). Routine prolactin measurement is not recommended because physiological changes do not reliably indicate tumor growth. Routine MRI is also not recommended; new neurological symptoms or objective visual deterioration should prompt neuro-ophthalmic assessment and MRI without gadolinium (1, 2, 9).

Confirmed symptomatic enlargement should first be treated by restarting a dopamine agonist (1, 9). The previously effective agent may be resumed: cabergoline is often favored for efficacy and tolerability, whereas bromocriptine has the longest accumulated pregnancy exposure (9, 10, 23). Transsphenoidal surgery is reserved for progressive visual or neurological compromise when medical treatment is ineffective or unsuitable, preferably during the second trimester; in late pregnancy, maternal status, fetal maturity, and delivery timing require multidisciplinary consideration (1, 2, 9).

Pituitary apoplexy requires urgent assessment, immediate stress-dose glucocorticoids when adrenal insufficiency is suspected, MRI without gadolinium, and multidisciplinary consultation (8, 9). Conservative management is appropriate when neurological and visual findings are stable; surgical decompression is considered for severe or progressive visual loss, declining consciousness, or failure of conservative treatment.

## 9 Postpartum reassessment and pathway re-entry

Breastfeeding is generally feasible when the tumor remained clinically stable and is not itself an indication to restart treatment (9, 24, 25). Dopamine agonists are usually deferred because they suppress lactation. If clinically significant regrowth, visual compromise, or other neurological risk develops, maternal safety takes priority; dopamine agonist therapy should be restarted and lactation discontinued when necessary (1, 2, 9).

Prolactinoma status should be reassessed after every pregnancy before routine treatment resumption (9). Symptoms should be reviewed postpartum; menstrual and gonadal recovery and prolactin are generally reassessed 3–6 months after delivery in nonlactating women or after breastfeeding ends. MRI is indicated for prior macroprolactinoma, symptoms or visual risk, suspected growth, or when imaging would alter treatment (1, 9). Prolactin measured during active lactation should not alone define remission.

Persistent hyperprolactinemia alone does not mandate treatment. Dopamine agonist therapy should be initiated or restarted when biochemical recurrence is accompanied by clinically meaningful hypogonadism or menstrual dysfunction, persistent bothersome symptoms, or MRI evidence of tumor persistence, growth, or mass-effect risk (1, 2, 9). Asymptomatic, eugonadal women with stable low-risk anatomy may remain under structured surveillance. Within the proposed framework, renewed near-term pregnancy intent prompts pathway A, an active endocrine or tumor-control indication prompts pathway B, and clinically stable low-risk disease prompts pathway C. Reported postpartum remission rates vary substantially across cohorts (approximately 41–68%); heterogeneity precludes applying a single probability to an individual patient (24–26).

## 10 Conclusion

Prolactinoma management in women of reproductive age should be dynamic and guideline-informed rather than governed by a fixed size-based algorithm. This Mini Review proposes an organizational framework, not a new clinical classification: diagnostic confidence precedes pathway assignment, reproductive and endocrine needs identify the dominant pathway, and tumor risk acts as a cross-cutting safety modifier. Dopamine agonists remain first-line, while surgery is appropriate for anatomically favorable tumors or when medication is ineffective, poorly tolerated, or unacceptable. Preconception care should restore ovulation and establish anatomical safety; pregnancy management is primarily symptom-driven. Postpartum reassessment should precede treatment resumption, because persistent hyperprolactinemia alone is not an indication for therapy; clinically meaningful hypogonadism, persistent symptoms, and tumor progression should guide re-initiation. Reassessment permits movement among fertility-driven, endocrine-stabilization, and surveillance-dominant pathways as reproductive goals, gonadal function, symptoms, and anatomy evolve. Prospective validation is required to establish the framework’s clinical utility.
